# Does the addition of fentanyl premedication impact brown fat uptake in children undergoing a warming protocol for FDG PET?

**DOI:** 10.1007/s00247-025-06381-5

**Published:** 2025-09-04

**Authors:** Mariama Lukulay, Pradipta Debnath, Christopher G. Anton, Yinan Li, Adam F. Prasanphanich, Susan E. Sharp, Bin Zhang, Andrew T. Trout, Cara E. Morin

**Affiliations:** 1 University of Cincinnati, Cincinnati, United States; 2 Cincinnati Childrens Hospital Medical Center, 3333 Burnet Ave, Cincinnati, OH 45229 USA

**Keywords:** Fentanyl, Positron emission tomography, Computed tomography

## Abstract

**Background:**

Fentanyl is used in some pediatric practices with a goal of suppressing 18F-fluorodeoxyglucose (18F-FDG) uptake in brown fat.

**Objective:**

The purpose of this study was to examine the frequency, intensity, and distribution of brown fat uptake in warmed children undergoing 18F-FDG PET/CT with and without premedication with fentanyl.

**Materials and Methods:**

This retrospective study included children (< 18 years old) who underwent 18F-FDG-PET from 2014 to 2024 at a center that routinely warms patients and uses intravenous fentanyl for brown fat suppression for most patients. Three radiologists assessed the presence, intensity, and location of brown fat uptake. Chi-square test and two-sample *t*-test were used to compare the demographics and brown fat uptake between premedication and non-premedication groups.

**Results:**

Among 873 18F-FDG-PETs, 595 (68%) were performed with fentanyl premedication and warming and 278 (32%) were conducted with warming alone. Brown fat uptake was observed in 46 (5.3%) FDG-PETs, 32/595 (5.4%) in the premedicated group and 14/278 (5.0%) in the non-premedicated group (*P* = 0.83). No differences were found in brown fat intensity or location based on premedication status. Age (14.5 vs. 8.5; *P* < 0.001) and BMI (20.1 vs. 17.7; *P* < 0.001) were significantly associated with brown fat uptake.

**Conclusion:**

Fentanyl premedication does not significantly affect brown fat uptake frequency, intensity, or location in warmed children undergoing 18F-FDG-PET.

**Graphical Abstract:**

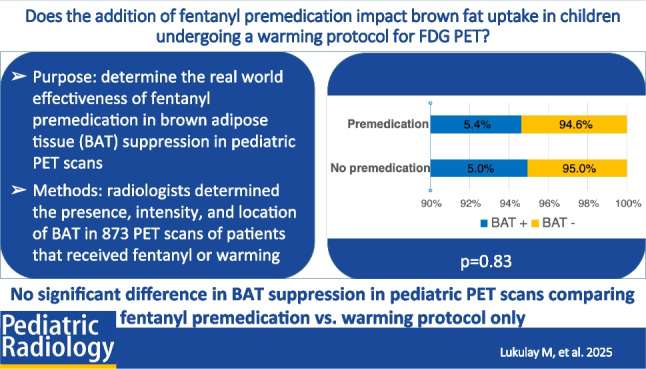

**Supplementary information:**

The online version contains supplementary material available at 10.1007/s00247-025-06381-5.

## Introduction

The use of ^18^F-fluorodeoxyglucose positron emission tomography/computed tomography (^18^F-FDG PET/CT or FDG PET) is well established for diagnosis, staging, and response assessment of cancers in children and young adults [1, 2]. However, interpretation of these scans can be complicated by the presence and metabolic activity of brown fat [1]. Brown fat has been shown to be especially prevalent in the younger population, usually located in the neck and supraclavicular regions, but also sometimes involving the mediastinum, paravertebral regions, and retroperitoneum [3, 4]. These anatomical sites overlap with regions commonly affected by malignancies (e.g., lymphoma), making it difficult to distinguish brown fat activity from pathologic FDG uptake [5].

To mitigate this diagnostic challenge, various strategies may be used to suppress the extent and intensity of FDG-uptake by brown fat. These include temperature control (e.g., warming) and pharmacologic interventions such as benzodiazepines, fentanyl, or beta blockers [3, 6–8]. Our institution uses a combined strategy of patient warming and pre-medication with fentanyl. However, not all patients receive fentanyl due to factors such as family preference or concurrent opioid administration.

This variation in clinical practice presents a valuable opportunity to further investigate the impact of fentanyl on brown fat suppression. The purpose of our study is to examine the frequency, intensity, and distribution of brown fat uptake in warmed children undergoing ^18^F-FDG PET/CT with and without premedication with fentanyl.

## Methods

This retrospective study was conducted at a quaternary academic pediatric center. Institutional review board (IRB) approval was obtained, along with a waiver of documentation of written informed consent. All study activities adhered to the guidelines of the Health Insurance Portability and Accountability Act (HIPAA).

Department and hospital electronic medical records were reviewed to identify all (pediatric) patients under the age of 18 years who underwent a clinically indicated ^18^F-FDG PET/CT between January 2014 and May 2024. To ensure an even age distribution and account for the known age dependence of brown fat uptake, a maximum of 50 scans per year of age was included. Examinations were excluded if performed at an outside hospital, if a non-FDG imaging agent was used, or if the primary diagnosis was pheochromocytoma. Pheochromocytoma is known to increase FDG-uptake in brown fat [9, 10].

Demographic and clinical data for each patient, including variables that may influence ^18^F-FDG metabolism (e.g., age, sex, BMI, concurrent medications, underlying diagnoses, glucose levels), were recorded along with details of patient preparation (e.g., fentanyl dose or reason for not administering). Concurrent medications administered within 24 h of the PET/CT exam were reviewed to identify children who received medications which may have suppressed brown fat uptake (e.g., opioids, benzodiazepines, or beta blockers). Additionally, the outside temperature on the day of each scan was documented.

All patients imaged with ^18^F-FDG PET/CT at our hospital undergo the following preparatory steps. First, patients are put in a warm room and/or in a warming chair. Then, warm blankets are placed around their entire body including wrapping around the legs and shoulders. All patients are warmed starting approximately 30–60 min prior to ^18^F-FDG-injection. A nursing workup is performed to determine if patients are appropriate for fentanyl administration. If deemed appropriate, fentanyl is administered 10 min prior to FDG injection at a dose of 1 mcg/kg up to 50 mcg/kg. For patients weighing more than 50 kg, fentanyl is administered on a sliding scale of 5 mcg per additional 10 kg of body weight beyond 50 kg, with a maximum dose of 70 mcg. Patients are monitored by nursing staff for at least 15 min after fentanyl administration.

Our institutional guidelines for fentanyl administration are as follows. Fentanyl is administered to individuals over 1 year of age with a diagnosis of lymphoma or those with head, neck, or upper extremity disease. In all other cases, fentanyl is administered in female individuals 1–25 years of age and male individuals 1–20 years of age. Administration is withheld under specific exclusion criteria. These include prior allergic reaction to fentanyl, driving self to appointment, body weight less than 10 kg, or patients less than 1 year of age. Fentanyl is also contraindicated in patients who have recently received narcotic medications, display current or potential respiratory compromise, or patient/family preference against fentanyl.

**Fig. 1:**
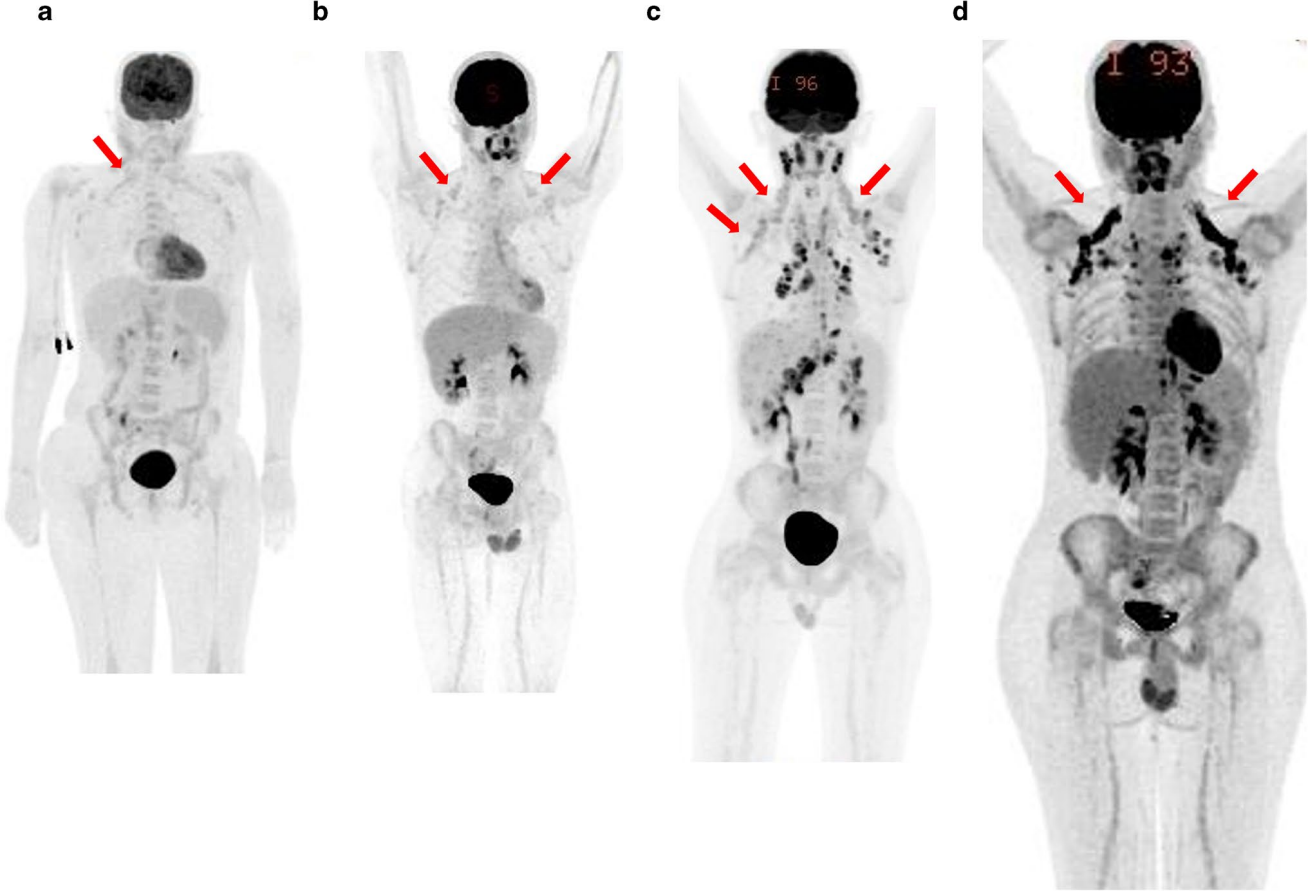
Examples of the Deauville-analog scoring system used to grade brown fat uptake intensity (*arrows*). All images are maximum uptake projection images from 18F-FDG-PET exams. **a** Score 2, uptake less than mediastinal blood pool; a 15-year-old female with thrombotic thrombocytopenic purpura; **b** score 3, uptake greater than mediastinal blood pool and less than liver; a 16-year-old male with suspected lymphoma; **c** score 4, uptake moderately more than liver; a 15-year-old male with suspected lymphoma; **d** score 5, uptake markedly more than liver; a 16-year-old male with Hodgkin lymphoma

Clinically obtained ^18^F-FDG PET/CT scans were reviewed by six board-certified pediatric radiologists, one of whom has additional board certification in nuclear radiology, with each scan reviewed by three radiologists. Reviewers assessed each scan for the presence, location, and intensity of brown fat uptake. Brown fat intensity was graded using a 5-point scale analogous to the Deauville scale (Fig. 1) [11]. The anatomical location of brown fat uptake was categorized into five predefined regions: neck, supraclavicular, paraspinal, mediastinal, and retroperitoneal/perinephric. Final determination of the presence of brown fat uptake for the purpose of study analysis was defined based on the agreement of at least two radiologists. Final anatomical distribution of brown fat uptake was based on the agreement of at least two radiologists. Final brown fat uptake intensity was defined by agreement of at least two radiologists or the mean of the scores of three radiologists if all disagreed.

### Statistical analysis

Participant demographics were summarized using descriptive statistics. Median and inter-quartile range (IQR) were used for continuous variables, whereas counts and percentages were used for categorical variables. Premedication and non-premedication groups were compared using the chi-square test (categorical variables) and the two-sample *t*-test (continuous variables). A statistically significant difference was defined as *P* < 0.05 for all inference testing. All statistical analyses were performed using MedCalc Statistical Software version 22.009 (MedCalc Software Ltd.) or R (R version 4.3.1, http://www.r-project.org).

## Results

A total of 873 ^18^F-FDG PET/CT scans were identified for analysis. Underlying diagnoses are summarized in Supplemental Table 1. Of the total sample, 595 patients (68.0%) received fentanyl (premedicated) and 278 patients (32.0%) did not (non-premedicated). Specific reasons for not administering fentanyl were documented in 111 (40%) reports, with the most common reasons being concurrent medications (*n* = 39, 35.1%), patient age (*n* = 20, 18.0%), and underlying medical condition (*n* = 17, 15.3%). Study sample demographics are summarized in Table 1. Non-premedicated patients were significantly younger and had lower height, weight, and BMI (*P* < 0.001) than premedicated patients. Brown fat uptake was present in a total of 46 patients (5.3%) (Table 2). Patients with brown fat uptake were significantly older and had significantly higher height, weight, and BMI. There was a trend toward lower outside temperature on the day patients with brown fat were scanned (*P* = 0.05).

**Table 1:** Demographics, clinical characteristics, and presence and intensity of brown fat uptake on ^18^F-FDG PET. Values are medians and inter-quartile ranges or counts and percentages. *P*-values reflect comparisons of the fentanyl and no fentanyl groups

**Demographics**	Entire sample (*n* = 873)	Non-premedicated with fentanyl (*n* = 278, 31.8%)	Premedicated with fentanyl (*n* = 595, 68.2%)	*P*-value
Age (years)	8.7 (4.3, 13.4)	6.0 (1.9, 12.1)	9.8 (5.9, 13.9)	< 0.001
Sex	F = 414 (47%) M = 459 (53%)	F = 139 (50%) M = 139 (50%)	F = 275 (46%) M = 320 (54%)	0.31
Height (cm)	132.3 (106.0, 158.0)	116.0 (81.5, 149.5)	139 (114, 161)	< 0.001
Weight (kg)	29 (18, 52)	21 (11, 40)	34.0 (21.0, 57.2)	< 0.001
BMI (kg/m^2^)	17.8 (16.0, 20.9)	17.3 (15.8, 19.9)	18.1 (16.1, 21.8)	0.002
Brown fat uptake present	46 (5.3%)	14 (5.0%)	32 (5.4%)	0.83
Brown fat uptake intensity	Score 2: 6 (13%) Score 3: 6 (13%) Score 4: 16 (35%) Score 5: 18 (39%)	Score 2: 3 (21%) Score 3: 3 (21%) Score 4: 3 (21%) Score 5: 5 (37%)	Score 2: 3 (9%) Score 3: 3 (9%) Score 4: 13 (41%) Score 5: 13 (41%)	0.34

**Table 2:** Relationship of selected clinical and environmental factors with the presence or absence of brown fat uptake (regardless of premedication group)

**Demographics**	No brown fat uptake (*n* = 827)	Brown fat uptake (*n* = 46)	*P*-value
Sex	Male = 441 (53%) Female = 386 (47%)	M = 18 (39%) F = 28 (61%)	0.06
Age (years)	8.5 (4.1, 13.1)	14.5 (9.5, 16.3)	< 0.001
Height (cm)	130.5 (105, 157)	159 (140, 169)	< 0.001
Weight (kg)	28 (18, 50.1)	54.2 (36.7, 65.2)	< 0.001
BMI (kg/m^2^)	17.7 (16.0, 20.8)	20.1 (17.8, 24.3)	< 0.001
Blood glucose (mg/dL)	89 (83, 95)	90 (86, 97)	0.13
Outside temperature (Fahrenheit)	56.5 (41.6, 70.1)	46.8 (34.1, 64.5)	0.05
Premedication (yes)	563 (68%)	32 (70%)	0.83

**Fig. 2:**
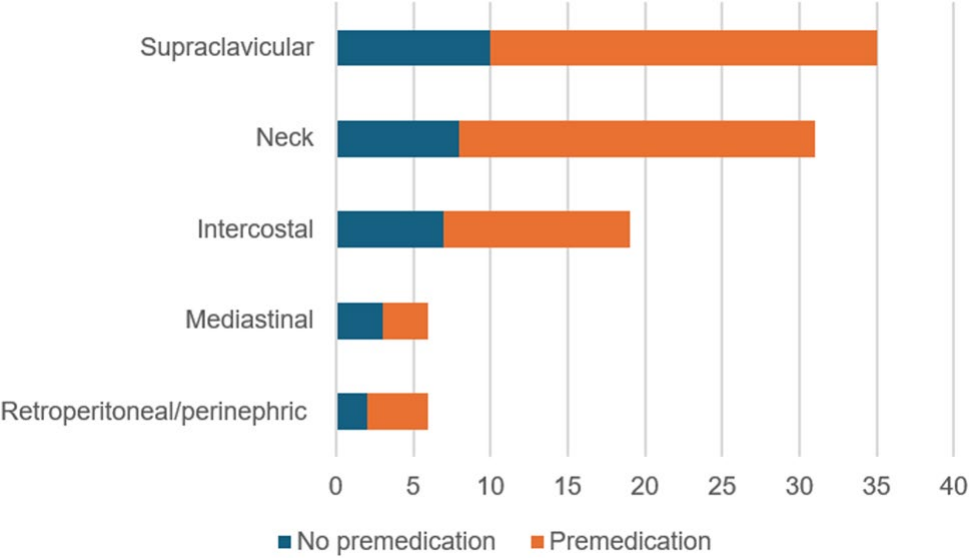
Distribution of locations of brown fat uptake found in patients with no premedication (*blue*) and those who received fentanyl premedication (*orange*)

There was no significant difference in the frequency of brown fat uptake between the premedicated and non-premedicated groups (*P* = 0.83). There was also no significant difference in the intensity of brown fat uptake between the premedicated and non-premedicated groups (*P* = 0.34). The supraclavicular region was the most common region involved by brown fat uptake in both groups (Fig. 2).

Among non-premedicated patients, 69/278 (24.8%) were receiving other medications that could suppress brown fat uptake (Supplemental Table 2). No children were receiving beta blockers. There was no significant difference in the frequency of brown fat uptake between children who received any concurrent medication and patients who were premedicated with fentanyl (Supplemental Tables 2 and 3). There was also no significant difference in the frequency of brown fat uptake between children who were not on concurrent medications and children who were premedicated with fentanyl.

## Discussion

In our retrospective real-world study of children undergoing ^18^F-FDG PET/CT, we found no significant difference in the frequency, distribution, or intensity of brown fat uptake between warmed pediatric patients who received or did not receive fentanyl premedication. The frequency of brown fat uptake was low in both groups at around 5%, which is substantially lower than previously reported frequencies of 30–50% in children [7, 8, 12]. This overall low frequency of brown fat uptake in our sample could be due to our warming strategy. Zukotynski et al. previously reported rates of 5–9% brown fat uptake in children placed in a 24-degree Celsius room for 30 min before and 1 h after FDG injection (vs. 27–31% brown fat uptake in children placed in a 21-degree Celsius room) with the greatest effect reported in summer and winter [13, 14]. However, others have found that pre-warming alone is not sufficient to prevent brown fat uptake in children [8]. These apparent contradictions may reflect differences in specific warming strategies among sites.

There is a paucity of research on suppression of brown fat uptake of ^18^F-FDG using fentanyl. Gelfand et al. reported the utility of fentanyl for suppressing brown fat uptake in children based on a retrospective review of pediatric and young adult patients undergoing ^18^F-FDG PET/CT. In that study, there was a significant reduction in brown fat uptake in 45 children premedicated with fentanyl when compared to 23 children who did not receive fentanyl (6.7% vs. 26.1%) and to 34 children who received low-dose diazepam (29.4%). Based on these results, fentanyl has been the premedication of choice at our institution. There was no mention of warming patients in the prior study by Gelfand et al., which reflects a meaningful difference in the preparation of our patient population who were all warmed and confounds direct comparison of results.

The mechanism by which fentanyl may suppress ^18^F-FDG uptake by brown fat is not well characterized. Paradoxically, in mechanistic studies of mice, fentanyl has been shown to activate brown fat as measured by sympathetic nerve activity and temperature, through a mechanism that involved activation of µ-opioid receptors in the brainstem [15, 16]. In these studies, the dose of fentanyl administered was 100 × the doses used in clinical practice for brown fat suppression; measurement of brown fat activity was based on physiology without correlation to ^18^F-FDG PET imaging, and, to our knowledge, similar studies have not been performed in humans.

Premedication with fentanyl is included among potential options for brown fat uptake suppression in the SNMMI Procedure Standard/EANM Practice Guideline on Pediatric 18F-FDG PET/CT for Oncology [1]. A recent international survey of pediatric nuclear medicine practitioners reported that almost a third utilize premedication in an effort to suppress brown fat uptake in children, with 4 out of the 43 respondent sites reporting use of fentanyl [17]. Among the other pharmacologic interventions available, Brady et al. previously demonstrated significantly lower frequencies of brown fat uptake in pediatric patients who received propranolol compared to those who were only warmed and compared to those who had no pre-PET preparation (9).

Our study identified significant associations between brown fat uptake and patient age and BMI, with older and larger patients showing the highest rates of brown fat uptake. These findings are consistent with prior research by Brady et al. [8], but contradict other literature [12, 18]. In contrast, we did not identify significant associations between brown fat uptake and sex or blood glucose levels. These findings align with those of Gelfand et al., who similarly reported no sex-based differences in brown fat uptake among pediatric patients [7]. However, multiple studies in adults have demonstrated higher brown fat uptake in females compared to males [19]. Our results show a trend toward an association between lower outdoor temperature and brown fat uptake, consistent with prior research which has suggested that colder environmental temperatures may influence brown fat activation [7, 8, 12].

Several limitations of the current study must be acknowledged. First, this study was conducted at a single academic institution, limiting generalizability. It is possible that preparation methods at our institution, including our warming protocol, account for the overall low rate of brown fat uptake and lack of a difference between the premedicated and non-premedicated groups. Second, our study was based on a convenience sample. Patients who were not administered fentanyl either declined fentanyl or had a reason for not receiving the medication, including concurrent opiate administration. The reason that fentanyl was not administered was available for less than 50% of patients in our sample and it is possible that unrecognized factors may have impacted the observed frequency of brown fat in this group. Our subanalysis based on concurrent medication administration failed to explain this effect but may be limited by sample size.

## Conclusion

Our retrospective real-world study of a convenience sample of pediatric patients undergoing ^18^F-FDG PET shows very low overall frequencies of brown fat uptake in warmed pediatric patients regardless of premedication. Furthermore, our study shows no significant difference in brown fat uptake of ^18^F-FDG between patients who received fentanyl for premedication and those who did not when all patients are warmed. These findings suggest that in the context of effective patient warming, the routine use of fentanyl for brown fat suppression may offer limited additional benefit. Given the need to balance potential benefits against the risks and side effects of pharmacologic interventions, our results support re-evaluating the necessity of premedication in warmed pediatric patients. Future prospective studies are warranted to more definitively assess whether premedication provides any additive value in this setting and to inform evidence-based practice guidelines.

## Supplementary information

Below is the link to the electronic supplementary material.

ESM1 (PDF 61.1 KB)

## Data Availability

No datasets were generated or analysed during the current study.
