# Supplementary material for: Does the addition of fentanyl premedication impact brown fat uptake in children undergoing a warming protocol for FDG PET?
Source: Pediatr Radiol. 2025 Sep 4;55(12):2643–50. doi: 10.1007/s00247-025-06381-5 (PMC12602624; doi:10.1007/s00247-025-06381-5)
Supplement: Supplementary file 1 — (PDF 61.1 KB) [file 247_2025_6381_MOESM1_ESM.pdf]

Supplemental Tables

Supplemental Table 1. Categories of FDG-PET indications.

Category	Count	Percent
Malignancy – Sarcoma	180	20%
Other/Unspecified	166	18.5%
Malignancy – Lymphoma	129	14.4%
LCH	82	9.1%
Malignancy – Neuroblastoma	70	7.8%
Malignancy – Leukemia	53	5.9%
Malignancy – Other/Unspecified	41	4.6%
Malignancy – Suspected	40	4.5%
Infection/Inflammation	27	3%
PTLD	26	2.9%
Malignancy – Head and Neck	24	2.7%
BMT/Transplant	15	1.7%
Malignancy – Hepatoblastoma	13	1.4%
Malignancy – Carcinoma	10	1.1%
Malignancy – Germ Cell Tumor	8	0.9%
Malignancy – Wilms	7	0.8%
Malignancy – Other 'blastoma'	5	0.6%
Malignancy – Retinoblastoma	2	0.2%
**Total**	**898**	**100%**

Supplemental Table 2. Frequency of administration of other medications that could impact brown adipose tissue (BAT) uptake, and frequency of BAT uptake among patients not premedicated with fentanyl (n = 278). Frequency of BAT uptake is compared to the frequency in patients who were premedicated with fentanyl (32/595, 5.4%).

Concurrent medication	Frequency	BAT uptake	P value compared to premedicated group
Opioid	50/278 (18%)	3/50 (6%)	0.75
Benzodiazepine	35/278 (13%)	0/35 (0%)	0.25
Opioid & benzodiazepine	16/278 (6%)	0/16 (0%)	>0.99
No opioid or benzodiazepine	209/278 (75%)	11/209 (5%)	0.95

Supplemental Table 3. Demographics, clinical characteristics, and presence and intensity of brown adipose tissue (BAT) uptake on ^{18}F-FDG PET

Demographics	Entire sample (n=873)	No medications (n=209)	Not premedicated with fentanyl; concurrent opioids/benzo (n=69)	Premedicated with fentanyl (n=595)	P-value
Age (years)	8.7 (4.3, 13.4)	5.8 (1.3, 12.0)	7.1 (3.0, 13.2)	9.8 (5.9, 13.9)	<0.001
Sex	F = 414 (47%)<br>M = 459 (53%)	F = 105 (50%)<br>M = 104 (50%)	F = 34 (49%)<br>M = 35 (51%)	F = 275 (46%)<br>M = 320 (54%)	0.58
Height (cm)	132.3 (106, 158)	113 (79, 147)	120 (94, 152)	139 (114, 161)	<0.001
Weight (kg)	29 (18, 52)	21 (10, 39)	24 (14, 51)	34.0 (21.0, 57.2)	<0.001
BMI (kg/m2)	17.8 (16.0, 20.9)	17. 1 (15.7, 19.6)	17.6 (16.3, 20.6)	18.1 (16.1, 21.8)	0.004

BAT uptake present	46 (5.3%)	11 (5.3%)	3 (4.4%)	32 (5.4%)	0.94
BAT uptake intensity	Score 2: 6 (13%)<br>Score 3: 6 (13%)<br>Score 4: 16 (35%)<br>Score 5: 18 (39%)	Score 2: 3 (27%)<br>Score 3: 1 (10%)<br>Score 4: 3 (27%)<br>Score 5: 4 (36%)	Score 2: 0 (0%)<br>Score 3: 2 (67%)<br>Score 4: 0 (0%)<br>Score 5: 1 (33%)	Score 2: 3 (9%)<br>Score 3: 3 (9%)<br>Score 4: 13 (41%)<br>Score 5: 13 (41%)	0.40
